# Poisoning by Plants

**Published:** 1875-08

**Authors:** 


POISONING BY PLANTS AND INSECTS.—A standing antidote for poison by oak, ivy, &c., is to take a handful of quicklime, dissolve it in water, let it stand half an hour, then paint the poisoned parts with it. Three or four applications will never fail to cure the most aggravated cases. Poison from bees, hornets, spider-bites, &c., is instantly arrested by the application of equal parts of common salt and bicarbonate of soda, well rubbed in on the place bitten or stung.

